# Perinatal Mortality in Eastern Uganda: A Community Based Prospective Cohort Study

**DOI:** 10.1371/journal.pone.0019674

**Published:** 2011-05-09

**Authors:** Victoria Nankabirwa, James K. Tumwine, Thorkild Tylleskär, Jolly Nankunda, Halvor Sommerfelt, for the PROMISE EBF Research Consortium

**Affiliations:** 1 Department of Paediatrics and Child Health, School of Medicine, College of Health Sciences, Makerere University, Kampala, Uganda; 2 Centre for International Health, University of Bergen, Bergen, Norway; 3 Division of Infectious Disease Control, Norwegian Institute of Public Health, Oslo, Norway; Tulane University, United States of America

## Abstract

**Background:**

To achieve a child mortality reduction according to millennium development goal 4, it is necessary to considerably reduce neonatal mortality. We report stillbirth and early neonatal mortality risks as well as determinants of perinatal mortality in Eastern Uganda.

**Methods:**

A community-based prospective cohort study was conducted between 2006 and 2008. A total of 835 pregnant women were followed up for pregnancy outcome and survival of their children until 7 days after delivery. Mother's residence, age, parity, bed net use and whether delivery took place at home were included in multivariable regression analyses to identify risk factors for perinatal death.

**Results:**

The stillbirth risk was 19 per 1,000 pregnancies and the early neonatal death risk 22 per 1,000 live births. Overall, the perinatal mortality risk was 41 [95%CI: 27, 54] per 1,000 pregnancies. Of the deaths, 47% followed complicated deliveries and 24% preterm births. Perinatal mortality was 63/1,000 pregnancies among teenage mothers, 76/1,000 pregnancies among nulliparous women and 61/1,000 pregnancies among women delivering at home who, after controlling for potential confounders, had a 3.7 (95%CI: 1.8, 7.4) times higher perinatal mortality than women who gave birth in a health facility. This association was considerably stronger among nulliparous women [RR 8.0 (95%CI: 2.9, 21.6)] than among women with a previous live birth [RR 1.8 (95%CI: 0.7, 4.5)]. All perinatal deaths occurred among women who did not sleep under a mosquito net. Women living in urban slums had a higher risk of losing their babies than those in rural areas [RR: 2.7 (95%CI: 1.4, 5.3)].

**Conclusion:**

Our findings strengthen arguments for ensuring that pregnant women have access to and use adequate delivery facilities and bed nets.

## Introduction

Low and middle income countries (LMIC) account for the vast majority of perinatal deaths, which includes stillbirths from 28 weeks of gestation and early (≤7 days after birth) neonatal deaths [1]. Ninety seven percent of an estimated yearly 3.3 million stillbirths and 2.8 million early neonatal deaths occur in these countries [1]. Documentation of perinatal deaths as well as identification of their major causes is a crucial first step for their prevention. However, information on deaths in most LMIC is scanty [1], [2], [3]. Methodological limitations, mostly associated with bias during recruitment, have undermined the credibility of results from hospital-based studies [4]. Available estimates are often based on retrospective household surveys, mainly demographic and health surveys (DHS), which are usually limited to prevalence estimates. This is also true for Uganda, that has a high estimated perinatal mortality of 36/1,000 pregnancies [5]. In addition, the risk factors and causes of perinatal mortality are poorly documented and understood. Presently, there is no published data on risk factors for perinatal deaths in Eastern Uganda. The objective of this prospective community-based cohort study was to estimate perinatal mortality and to identify the main risk factors of perinatal death in Mbale, Eastern Uganda.

## Methods

We obtained written informed consent from each study participant. Ethical approval was obtained from the Makerere University Research and Ethics Committee, the Uganda National Council for Science and Technology, and from the Regional Committee for Medical and Research Ethics for Western Norway (REK VEST, approval number 05/8197).

The study was undertaken during the cluster-randomized PROMISE EBF intervention trial, where we promoted exclusive breastfeeding by individual peer counselling in the intervention areas (Clinical trials gov: NCT00397150) [6]. Data collection for this study started in Uganda in January 2006 and ended in May 2008.

### Study site

The study was conducted in Mbale district which had an estimated population of 720,000 [7] and is located 300 km North-East of Kampala. The study area is served by Mbale Hospital, which doubles as the district and regional referral hospital. The HIV prevalence among pregnant women in antenatal clinics in Mbale was approximately 5–6% during the study period. Most of the people living in the area were subsistence farmers. Mbale district comprised of 7 counties; the study was conducted in the two biggest counties, namely Bungokho County (rural) and Mbale Municipality (urban). Twenty four clusters were included in the study, 18 rural and 6 urban. Six clusters in Mbale municipality were selected from all its three municipal divisions. Most of the urban areas were large informal settlements. Eighteen clusters in Bugonkho County were chosen from eight out of its eleven sub-counties. Clusters were included if they neighboured the main road out from Mbale Municipality or were on the 1^st^ or 2^nd^ branch off the main road, had a population of at least 1,000 inhabitants and represented a social and administrative unit.

### Study subjects

Between January 2006 and September 2007, all pregnant women in the selected clusters were approached by the study team. They were eligible if they resided in the study area, were seven or more months pregnant, opted to breastfeed their infants and consented to participate in the study. Women were excluded if they had an intention to leave the area during the study period. Both singletons and twin deliveries were included in the study. In the PROMISE-EBF trial, 886 pregnant women were identified and approached. Of these, 875 (99%) accepted to participate in the study. Of the 875, 12 (1%) women did not meet the eligibility criteria and 28 (3%) relocated out of the study area after recruitment but before the endpoint of 7 days after delivery and were lost to follow-up. We analyzed data from the remaining 835 women.

### Data collection

At recruitment, trained data collectors fluent in the local language, Lumasaaba, administered a pre-tested structured Lumasaaba questionnaire. Information was collected on socio-demographic characteristics, antenatal care attendance, marital status and main source of income. Information was also collected on the current pregnancy and use of bed nets. The women were followed up through the pregnancy until 6 months postpartum. All births, deaths and details of the delivery were recorded within four weeks of delivery. For the perinatal deaths, a standard World Health Organization (WHO) verbal autopsy questionnaire was used to collect information for a standard algorithm determining the likely cause of death [8]. The questionnaire had both an open-ended section for reporting verbatim and a closed-ended section with filter questions. The verbal autopsies were done as soon as socially acceptable (2 weeks to 2 months after the loss of the baby).

### Statistical analysis

Using EpiHandy software (www.openXdata.org, version 165.528-142 RC) on handheld computers, the data was entered in the field. We undertook data analysis using Stata version 9 (StataCorp LP, TX, U.S.). Continuous variables were categorized to avoid doubtful assumptions about linearity. The primary outcome was perinatal death, defined as pregnancy loss occurring after seven completed months of gestation (still birth) or deaths within the first seven days of delivery of a live born child (early neonatal death), its confidence limits calculated using the exact method. Secondary outcomes included stillbirths and early neonatal deaths. The stillbirth risk was defined as the number of babies born dead after 28 weeks of gestation per 1,000 pregnancies and the early neonatal mortality risk was defined as the number of deaths in the first 7 days of life per 1,000 live births. The exposure variables were maternal age, parity, mother's education, place of delivery, antenatal care attendance, marital status, residence, household wealth index, and use of bed nets. Crude risk ratios (RR) and 95% confidence intervals were estimated for the exposure variables. We used multivariable generalized linear model (GLM) regression analysis with a log link to estimate the adjusted RR of the independent variables on perinatal mortality. Initially, all these variables were included in the crude analyses. However, only variables that were associated with perinatal deaths yielding a P-value <0.2 were retained in the model.

We calculated the difference in perinatal mortality between women delivering at home and in health facilities or with a traditional birth attendant. We also calculated the perinatal mortality difference for sleeping under a mosquito net; the corresponding RR could not be calculated because no perinatal deaths were recorded in this category.

### Definitions

We categorized marital status into two categories: ‘married’ and ‘un-married’. The unmarried category comprised cohabiting, single, divorced, widowed and separated. We categorised place of delivery into two groups: ‘hospital/clinic/local maternity/traditional birth attendant's place or on the way to hospital’, and ‘at home’, i.e. without a skilled birth attendant. We defined parity according to the number of previous live births and grouped education into two categories: ‘less than or equal to 7 years of education’ and ‘more than 7 years of education’. To measure bed net use, mothers were asked whether or not they slept under a bed net.

We created a composite index of wealth (socio-economic status) using multiple correspondence analysis (MCA). Because the MCA technique allows combination and ranking of a large number of variables into smaller and fewer variables without prejudgment, it is considered a more accurate indicator of socioeconomic status (SES) than single items such as occupation or possession of particular items. Also, in comparison to principal component analysis, the MCA technique is more appropriate for discrete variables. This was important in this study because several relevant variables could only be categorical. Furthermore, unlike principal component analysis, which clusters variables together, MCA clusters the categories within these variables together. We used MCA on possession of a TV, radio, mobile phone, chair, cupboard, refrigerator, type of toilet, type of house walls as well as electricity and water source in the home. We used dates of the last menstrual period to estimate gestational age. Preterm births were infants born less than 37 weeks of gestation. Complicated deliveries were those associated with haemorrhage, cord complications, obstructed, prolonged labour, pre-eclampsia, eclampsia, and breech or other non-cephalic presentations.

## Results

In our cohort of 835 pregnant women, the average age was 25 (ranging from 14 to 44) years. They had a mean of 7 (range 0 to 18) years of formal education. Their mean and median parity was 3, ranging from 0 to 14, with an inter-quartile range of 3 to 5. Socio-demographic factors are presented in Table 1.

**Table 1:** Risk factors stillbirths and early neonatal deaths in a cohort of 835 women in Mbale, Eastern Uganda.

	Births (N = 835)	Stillbirths (N = 835)		Early neonatal deaths (N = 819)	
Variable	N (%)	Unadjusted RR (95% CI)	Adjusted RR (95% CI)	Unadjusted RR (95% CI)	Adjusted RR (95% CI)
Maternal age					
<20 years	190 (23)	5.8 (1.8, 18.9)	3.1 (0.9, 10.7)	0.8 (0.2, 2.9)	0.8 (0.2, 3.2)
20–30	489 (58)	1	1	1	1
>30	156 (19)	2.4 (0.5, 10.4)	4.0 (0.8, 18.5)	1.6 (0.6, 4.6)	2.0 (0.7, 6.0)
Parity					
0	211 (25)	6.5 (2.3, 18.5)	7.2 (2.0, 25.5)	1.2 (0.4, 3.3)	1.8 (0.6, 5.6)
≥1	624 (75)	1	1	1	1
Mother's education					
≤7 Years	591 (71)	1.2 (0.4, 3.8)	1.1 (0.3, 3.5)	0.7 (0.3, 1.7)	0.6 (0.2, 1.6)
> 7 Years	244 (29)	1	1	1	1
Residence					
Urban	190 (23)	2.0 (0.7, 5.5)	2.9 (1.1, 7.7)	2.2 (0.9, 5.6)	2.5 (1.0, 6.6)
Rural	645 (77)	1	1		1
Marital status					
Unmarried	513 (61)	1.6 (0.6, 4.2)		1.0 (0.4, 2.6)	
Married	322 (39)	1		1	
Household wealth index					
Bottom 20%	167 (20)	1.6 (0.4, 5.9)		1.7 (0.6, 5.1)	
Middle 40%	333 (40)	1.4 (0.5, 4.4)		0.7 (0.2, 2.3)	
Top 40%	335 (40)	1		1	
Antenatal care attendance					
No	235 (28)	1.5 (0.6, 4.2)		1.3 (0.5, 3.4)	
Yes	600 (72)	1			
Use of mosquito bed nets					
No	484 (58)				
Yes	351 (42)				
Place of delivery					
Home	345 (41)	2.4 (0.9, 6.5)	4.2 (1.5, 12.1)	2.3 (0.9, 5.8)	3.1 (1.2, 8.5)
Hospital/clinic/traditional birth attendant†	490 (59)	1	1		1
Complicated delivery					
Yes	80 (10)	3.1 (1.0, 9.5)		34.2 (11.6, 101.3)	
No	755 (90)	1		1	
EBF					
Intervention	433 (52)	1.1 (0.4, 2.8)		0.7 (0.3, 1.8)	
Non intervention	402 (48)	1		1	

*CI indicates confidence interval.

† Hospital/clinic/traditional birth attendant includes 474 women who delivered at a health facility and 16 women that delivered with a traditional birth attendant (TBA). There were no deaths in the latter group.

Out of the 835 pregnancies, there were 17 twin births and 818 single deliveries. There were two maternal and 34 perinatal deaths. Nearly half of the deaths were associated with a complicated delivery, which was associated with an unadjusted 10.6-fold higher mortality risk (table 2).Likely conditions associated with the deaths and indentified via verbal autopsies included: cord prolapse, obstructed labour, antepartum haemorrhage, mal-presentations, preterm births, fever/malaria in pregnancy, neonatal tetanus and preeclampsia (table 3). None of the perinatal deaths that occurred at home were registered anywhere and there was no death certificate available for any of the deaths, including those that occurred in hospital.

**Table 2:** Number of perinatal deaths, mortality risk by risk factor and risk ratios for perinatal death, in a cohort of 835 women in Mbale, Eastern Uganda.

	Perinatal deaths (N = 835)			
Variable	Perinatal deaths (N = 34)	Perinatal death risk/1,000 pregnancies	Unadjusted RR (95% CI)	Adjusted RR (95% CI)
Maternal age				
<20 years	12	63	2.2 (1.0, 4.7)	1.7 (0.8, 3.8)
20–30	14	29	1	1
>30	8	51	1.8 (0.8, 4.2)	2.5 (1.0, 5.9)
Parity				
0	16	76	2.6 (1.4, 5.0)	3.3 (1.5, 7.0)
≥1	18	29	1	1
Mother's education				
≤7 Years	23	39	0.9 (0.4, 1.7)	0.8 (0.4, 1.6)
>7 Years	11	45	1	1
Residence				
Urban	13	68	2.1 (1.1, 4.1)	2.7 (1.4, 5.3)
Rural	21	33	1	1
Marital status				
Unmarried	19	37	1.3 (0.6, 2.4)	
Married	15	47	1	
Household wealth index				
Bottom 20%	10	60	1.7 (0.7, 3.8)	
Middle 40%	12	36	1.0 (0.5, 2.2)	
Top 40%	12	36	1	
Antenatal care attendance				
No	12	51	1.4 (0.7, 2.8)	
Yes	22	37	1	
Use of mosquito bed nets				
No	34	70		
Yes	0	0		
Place of delivery				
Home	21	61	2.3 (1.2, 4.5)	3.7 (1.8, 7.6)
Hospital/clinic/traditional birth attendant†	13	27	1	1
Complicated delivery				
Yes	18	225	10.6 (5.6, 20)	
No	16	21	1	
EBF				
Intervention	19	44	0.9 (0.4, 1.7)	
Non intervention	15	37	1	

**Table 3:** Summary characteristics of the 34 perinatal deaths from a cohort of 835 women in Mbale, Eastern Uganda.

Likely cause of death	Place of delivery	Type of perinatal death	Number
Ante-partum haemorrhage	Home	Early neonatal death	2
Breech Presentation	Home	Still birth	1
Breech presentation	Home	Early neonatal death	1
Fever/Malaria	Home	Still birth	3
Fever/Malaria	Hospital	Early neonatal death	2
Mal presentation	Hospital	Early neonatal death	1
Malaria/Preterm birth	Hospital	Still birth	3
Not determined	Hospital	Still birth	1
Not determined	Hospital	Early neonatal death	1
Obstructed labour	Hospital	Still birth	1
Obstructed labour	Home	Early neonatal death	2
Obstructed labour	Hospital	Early neonatal death	1
Preeclampsia	Hospital	Still birth	1
Preterm birth	Home	Still birth	4
Preterm birth	Hospital	Still birth	1
Prolapsed cord	Home	Still birth	5
Prolapsed cord	Home	Early neonatal death	1
Prolonged labour	Hospital	Early neonatal death	2
Tetanus	Home	Early neonatal death	1

### Stillbirths

Out of the 34 perinatal deaths, 16 (47%) were stillbirths, corresponding to a stillbirth risk of 19 (95% CI: 11, 31) per 1,000 pregnancies (table 1). We recorded no stillbirths among the multiple pregnancies. Nulliparous women were in the adjusted analysis seven times more likely to have a stillbirth than women with a prior live birth. Other factors associated with stillbirths were delivering at home and living in an urban area (table 1). The excess stillbirth risk, attributable to not sleeping under a bed net was 33 (95% CI: 17, 49) per 1,000.

### Early neonatal deaths

There were 18 early neonatal deaths, constituting 53% of all the perinatal deaths. The early neonatal mortality risk was 22 (95% CI: 13, 35) per 1,000 live births. Eighty one percent of the deaths occurred on the day of birth. Women who delivered at home were 3 times more likely to have an early neonatal death compared to women who delivered in a health facility. The risk of early neonatal death was 2.5 times higher for women in urban areas compared to those in rural areas. The excess early neonatal death risk attributable to not sleeping under bed nets was 38 (95% CI: 21, 56) per 1,000.

### Perinatal deaths

None of the women who used mosquito bed nets experienced a perinatal death. The excess unadjusted perinatal mortality attributable to not using a bed net was 70 (95% CI: 47, 93) per 1,000. After adjusting for place of delivery (a proxy for health seeking behaviour), the excess risk was 71 (95% CI: 48, 93) per 1,000. This excess risk was substantially higher in nulliparous women than in women with a previous live birth [risk difference 134 (95% CI: 73, 196) per 1,000 versus 49 (95% CI: 27, 72) per 1,000]; P-value for homogeneity of risk differences = 0.010.

In the adjusted analysis (Table 2), women who delivered at home were 3.7 times more likely to experience a perinatal death than those who delivered in a health facility or under the guidance of a traditional birth attendant. This association was substantially stronger among nulliparous women [RR: 8.0 (95% CI: 2.9, 21.6)] than among those with a previous live birth [RR: 1.8(95% CI: 0.7, 4.5)], p-value for RR homogeneity = 0.029. The adjusted difference in the perinatal death risk between home and an alternative place of delivery was also substantially stronger among nulliparous women [adjusted risk difference 159 (95% CI: 53, 264) per 1,000] than among women with a previous live birth [adjusted risk difference 7 (95% CI: −11, 25) per 1,000], P-value for homogeneity of risk differences = 0.006. Delivering at home was associated with stillbirths [RR: 4.2 (95% CI: 1.5, 12.1)] as well as with early neonatal deaths [RR: 3.1 (95% CI: 1.2, 8.5)] in adjusted analysis (table 1).

The adjusted perinatal mortality risk among nulliparous women was 3.3 times higher than in woman who had given birth to a live baby in a prior pregnancy, most of this difference being attributed to the association between parity and stillbirth risk (Table 2).

Most of the urban areas in this study were slums and most deaths occurred here rather than in the rural areas. Perinatal mortality was 68/1,000 pregnancies in urban areas in comparison to 33/1,000 in rural areas. Thus, women in the urban areas had a 2.7 times higher adjusted risk of losing their babies in the perinatal period compared to those in the rural areas. Taking the design effect of the PROMISE-EBF clusters into account had no effect on the RR and a negligible effect on precision.

## Discussion

This study describes high perinatal risks in Mbale, Eastern Uganda: the stillbirth risk was 19 per 1,000 pregnancies, the early neonatal death risk 22 per 1,000 live births and the perinatal mortality risk 41 per 1,000 pregnancies. It is probable that the risks are higher in the district as a whole because we purposely chose clusters that were near main roads and therefore had better access to health facilities compared to some of the excluded clusters. However, our estimate for perinatal mortality risk is in line with the national estimate of 36/1,000 pregnancies in Uganda [5], [9], the Sub-Saharan African regional estimates, but much higher than what is reported in high-income countries in Europe (8/1,000) and North America (7/1,000) [9]. In 2008, neonatal deaths constituted 21% while HIV constituted 5% of an estimated 189,990 under five deaths in Uganda [9].

In addition, the study describes probable risk factors for stillbirths, early neonatal deaths and perinatal deaths, the most prominent being home delivery, In a review of 40 DHSs with complete data, almost half of all the perinatal deaths occurred after a home delivery [3]. In the current study, home delivery was an important risk factor for stillbirths, early neonatal deaths and perinatal deaths. Women who delivered at home were 4 times more likely to have a stillbirth and 3 times more likely to have an early neonatal death. This is consistent with findings elsewhere [10]. Nulliparity increased the risk of perinatal deaths 3-fold, most of this being explained by the increase in stillbirth risk.

There was significant effect measure modification (interaction) on both the additive and multiplicative scales between place of delivery and parity as risk factors for perinatal death. Thus, the increase in perinatal mortality associated with home deliveries was substantially higher for nulliparous women than for women with a previous live birth. Assuming that we in our analyses have managed to adjust for relevant confounders, and because we believe that the health facilities were capable of managing many of the life threatening conditions seen in our cohort, we estimate that one perinatal death would be averted for every 6 (95% CI: 4, 18) nulliparous women who delivered in a health facility rather than at home. In our cohort alone with 57 nulliparous women giving birth at home, this would have meant 9–10 children saved. This could be an underestimate because some of the women who lost their babies in hospital initially tried to deliver at home and only went to the hospital when it was too late to save the child.

There were no stillbirths or early neonatal deaths recorded among women who slept under mosquito bed nets. The risk of perinatal death associated with not sleeping under a bed net was particularly high among nulliparous women. Again assuming that we in our analyses successfully adjusted for relevant confounders, we estimate that one perinatal death was averted for every 7 (95% CI: 15, 14) nulliparous women who slept under a bed-net. It is generally accepted that insecticide treated bed nets reduce malaria transmission and decrease mortality [11] but evidence on the impact of bed nets on perinatal mortality has been mixed [12]. Malaria in pregnancy damages the foetal placental unit and it has been shown to lower the mean birth weight by an average of approximately 150 g in primigravidae [13], [14]. Low birth weight in turn, predisposes to perinatal mortality [15]. Similar to our findings, a systematic review of six randomized trials, four of which were from Sub-Saharan Africa, concluded that mosquito bed nets reduced foetal loss in the first to fourth pregnancy [16]. On the other hand, a Kenyan trial in which a rural population was randomized to receive or not to receive insecticide-treated bed-nets found no difference in perinatal deaths between the intervention and control arms.

Nearly one fourth of the stillbirths and early neonatal deaths in our study reportedly occurred following a preterm birth. In addition, nearly half of the births were complicated by haemorrhage, cord complication or breech or other non-cephalic presentations, and these were likely to have contributed to late foetal and early neonatal death. Similar findings have been made elsewhere. This indicates that perinatal deaths arise from factors occurring during pregnancy and delivery. In our study, there was a substantially higher risk of stillbirths, early neonatal death and thereby perinatal death in urban slums than in rural areas. Similarly, a study in the urban slums of Nairobi found very high mortality, particularly in the perinatal period [17].

Not surprisingly, none of the deaths that occurred at home were recorded in any formal system and none, including those that occurred in health facilities, had a death certificate. Many of these deaths would therefore have been missed by facility-based studies and the perinatal deaths of mothers dying during or shortly after delivery would also have been missed by retrospective surveys which are the principle sources of data on perinatal mortality in low and middle income countries. This prospective study was able to take into account all these factors, and provide a comprehensive description of likely risk factors for perinatal death.

The distinction between early neonatal deaths and still births is difficult, especially in deliveries that occur at home where there are no skilled health workers who can identify faint signs of life [5]. It is probable that some early neonatal deaths could have been misclassified as stillbirths [10]. Yet, the proportion of stillbirths and early neonatal deaths in our study is in line with WHO estimates for East Africa [18].

Verbal autopsies were used in this study to ascertain likely causes of stillbirths and early neonatal deaths. Though verbal autopsies are generally accepted in many countries without vital registration, their validity and sensitivity varies widely between locations [19]. In this study, we used a standard WHO verbal autopsy tool that had been validated in Uganda [8].

Perinatal mortality is an important public health problem in Uganda and other LMIC. In the current study, perinatal deaths were associated with preterm births, malaria in pregnancy and complicated deliveries. The main preventable risk factors were delivering at home and not sleeping under a bed net, especially for women who previously had no live born child. Living in an urban informal settlement also carried an increased risk for stillbirths, early neonatal deaths and perinatal deaths. Our findings strengthen arguments for ensuring that pregnant women have access to and use adequate delivery facilities and bed nets. Further studies are required to measure the degree to which increasing bed net usage and health facility delivery will reduce perinatal mortality, especially among nulliparous women.
